# Beyond frictionless access: a testable downstream accountability framework for the WHO pathogen access and benefit-sharing annex

**DOI:** 10.7189/jogh.16.03036

**Published:** 2026-11-13

**Authors:** Qi Shao

**Affiliations:** Department of Situation and Policy, Huaibei Normal University, Huaibei, China

**Keywords:** WHO Pandemic Agreement, Pathogen Access and Benefit-Sharing, pandemic preparedness, pathogen sharing, benefit sharing, global health governance, equity, access and benefit-sharing

## Abstract

The World Health Organization (WHO) Pandemic Agreement depends on a workable Pathogen Access and Benefit-Sharing (PABS) annex that can reconcile rapid pathogen sharing with equitable access to pandemic-related benefits. This viewpoint presents a testable downstream accountability proposal rather than a description of agreed PABS rules. Article 12 already establishes the PABS system, requires the annex to address traceability, provides for legally binding contracts with participating manufacturers, and sets a target for WHO access to 20% of real-time production during a pandemic emergency. The proposal developed here adds an operational downstream trigger, persistent provenance identifiers, pass-through contractual duties, transparent compliance review, and pooled allocation. Routine public-health and non-commercial scientific access would remain rapid and low-friction, and obligations would arise when documented PABS use enters defined product-oriented, intellectual-property, regulatory, licensing, manufacturing, or commercial activity. Persistent identifiers would document provenance, not legal causation, while pooled benefit sharing would avoid sequence-by-sequence valuation. The model also acknowledges unresolved questions of legal authority, national access and benefit-sharing law, allocation governance, non-participating manufacturers, and political acceptability that require negotiation and prospective testing.

The World Health Organization (WHO) Pandemic Agreement represents a major effort to strengthen global preparedness after the inequities exposed during COVID-19 [1]. Rapid scientific innovation did not automatically translate into equitable access to countermeasures, particularly for countries with limited manufacturing capacity and purchasing power [2–4]. The underlying governance problem persists: pathogen sharing requires international cooperation, but cooperation may weaken if countries providing biological resources and information do not trust that resulting benefits will be shared fairly.

Article 12 already resolves some elements of that bargain. It establishes a multilateral Pathogen Access and Benefit-Sharing (PABS) system, places access and benefit sharing on an equal footing, requires the annex to address traceability and open data, and provides for legally binding contracts with participating manufacturers, including a target for WHO access to 20% of real-time production during a pandemic emergency [1]. It also requires consistency with the objectives of the Convention on Biological Diversity and the Nagoya Protocol [5,6], and provides that parties should review and, as appropriate, align relevant national or regional access and benefit-sharing measures [1]. However, the operational architecture remains under negotiation. In July 2026, the seventh Intergovernmental Working Group (IGWG) meeting continued discussions on access, benefit-sharing, contracts, laboratory networks, recognised sequence databases, and overall PABS architecture [7].

The presented framework in this viewpoint therefore does not treat persistent identifiers, a downstream trigger test, a standardised contract architecture, a public compliance registry, independent technical review, graduated contractual remedies, or a contribution-sensitive pooled allocation formula as agreed WHO rules. The central question is where accountability can be placed without slowing routine scientific exchange.

## MOVING COMPLIANCE DOWNSTREAM

Requiring transaction-level permission, attribution, or payment for every transfer, database query, or analytical reuse could fragment data flows and impede the large-scale comparisons on which pathogen surveillance depends [8–10]. A downstream model instead preserves rapid access for surveillance, risk assessment, and non-commercial public-health research while attaching additional PABS obligations to defined value-creating activities (**Figure 1**).

**Figure 1:**
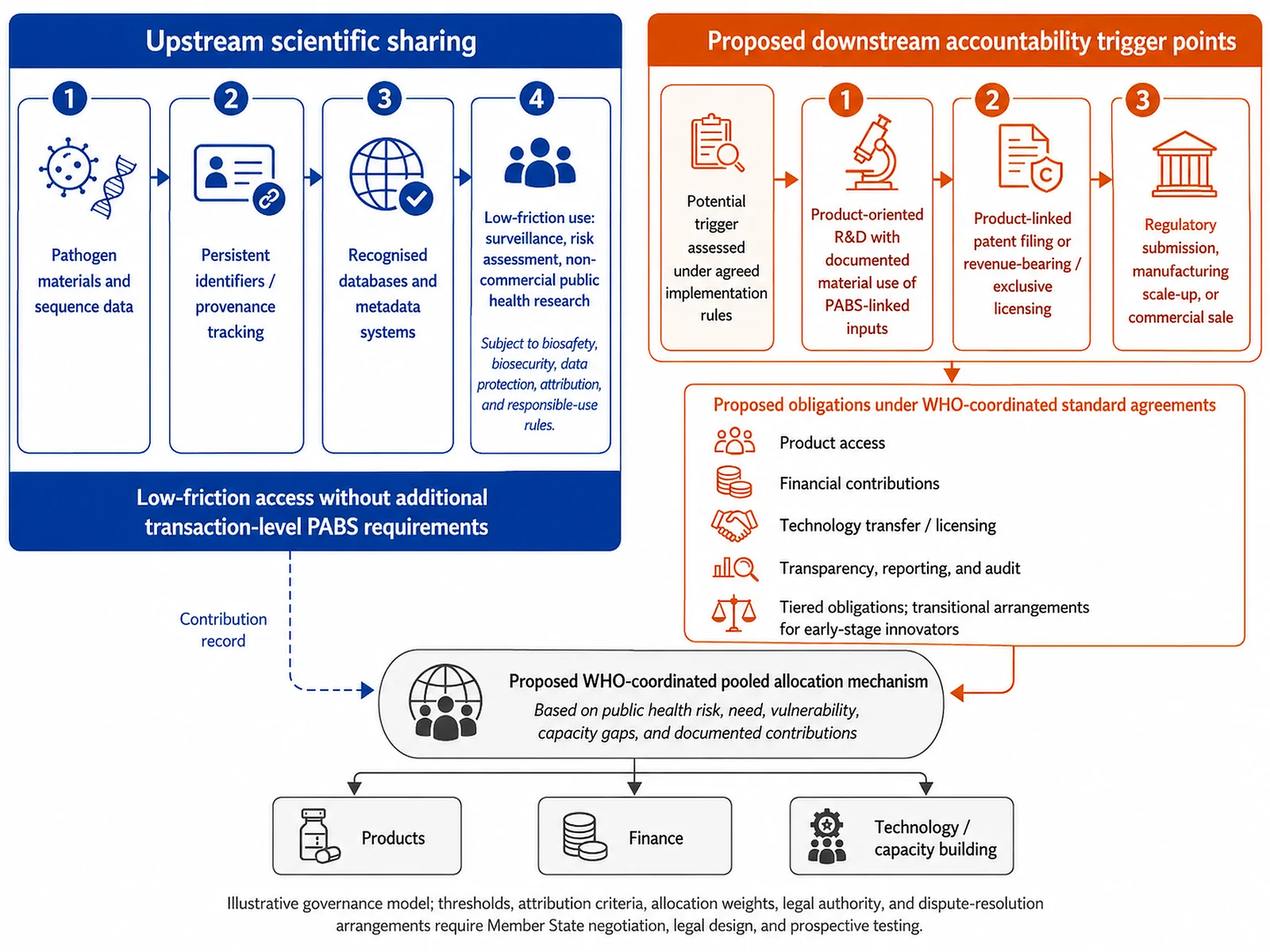
A downstream accountability framework for the WHO PABS system. The framework separates low-friction public-health and non-commercial scientific access from proposed downstream accountability triggers and obligations. Persistent identifiers document provenance but do not establish legal causation or quantify contribution. Illustrative triggers include product-oriented research and development involving documented material use of PABS-linked inputs, product-linked patent filing or revenue-bearing or exclusive licensing, and regulatory submission, manufacturing scale-up, or commercial sale. Contribution records may inform pooled benefit allocation without sequence-by-sequence valuation. Thresholds, attribution criteria, allocation weights, legal authority, and dispute-resolution arrangements require Member State negotiation, legal design, and prospective testing. PABS – Pathogen Access and Benefit-Sharing; R&D – research and development; WHO – World Health Organization.

A practical trigger should depend on documented use in a covered activity, not proof that one sample or sequence legally caused a final product. Under the proposed model, full obligations would begin when PABS-linked inputs are materially used in product-oriented preclinical or clinical development, a product-linked patent filing, a regulatory submission, a revenue-bearing or exclusive licence, manufacturing scale-up, or commercial sale. Incidental database exposure or general background use would not suffice. Public-private collaborations and early translational projects would enter a notification stage when product-directed work or commercial rights arise, with full duties deferred until a defined trigger. A university would remain in the low-friction layer for ordinary academic work, but a spin-out, licence, or product-directed industry agreement could activate downstream duties.

Persistent identifiers would record provenance across repositories, contractors, artificial-intelligence workflows, and subsequent licences, but would not establish legal causation or quantify contribution. The proposed rule asks a narrower question: was a PABS-linked input materially used in a covered activity? Standard clauses could pass relevant duties and provenance metadata to contract research organisations, subcontractors, platform companies, repositories, and licensees. For large training data sets, the trigger would depend on documented inclusion of PABS-linked data in a product-directed workflow rather than a numerical causal share for each sequence.

This approach would complement rather than replace national access and benefit-sharing law. Article 12 recognises state sovereignty, requires consistency with the objectives of the Convention on Biological Diversity and the Nagoya Protocol, and anticipates that parties will review and, as appropriate, align relevant national or regional access and benefit-sharing measures so that contrary, inconsistent, or duplicative measures are not applied once all elements of the PABS system enter into operation [1,5,6,11,12]. Member States would still need to define PABS scope in domestic law and clarify sequence information. A PABS coverage certificate could be tested as evidence that a transaction falls within the multilateral system, but such a safe harbour would require intergovernmental agreement.

## MAKE BENEFIT SHARING AUTOMATIC AND VERIFIABLE

Participating manufacturers should sign standard WHO contracts before an emergency, building on the legally binding contracts contemplated by Article 12 [1]. Minimum clauses could define triggers, monetary and product commitments, reporting, audit, confidentiality, pass-through duties, governing law, dispute procedures, and remedies. Obligations could be tiered by turnover, product type, manufacturing capacity, and commercial significance, with transitional arrangements for early-stage innovators. This proposal does not grant WHO unilateral coercive authority: contracts bind their parties, and WHO could not simply compel a manufacturer operating outside the PABS contracting system. Closing that gap would require participation incentives and, where Member States agree, domestic legal or procurement measures.

The headline 20% product-access target also requires operational definitions [1]. Real-time production should be measured through auditable output metrics, while affordability should consider production costs, public subsidies, country income, and scaling capacity. COVID-19 showed that collaboration and manufacturing capacity did not by themselves ensure equitable access [2–4]. Under the proposed model, the WHO-mediated 20% target could serve as a minimum emergency benchmark rather than a ceiling on additional measures.

Transparency and compliance mechanisms are also proposals subject to legal design. WHO could maintain a public registry of participating manufacturers, contracted commitments, aggregate deliveries, monetary contributions, and verified performance while protecting genuinely confidential technical information. An independent panel could review evidence and publish findings. Remedies should be limited to those lawfully available under the governing contract or applicable procurement rules, such as corrective plans, recovery of unpaid sums, publication of verified non-compliance, or proportionate restrictions on future WHO-linked contractual opportunities where expressly authorised. Cross-jurisdictional enforceability, due process, audit resources, and non-participants remain implementation constraints.

## POOLING BENEFITS WHILE RECOGNISING CONTRIBUTION

Assigning each product to a single country or sequence would be scientifically artificial and administratively costly. Countermeasures commonly emerge from multiple samples, genomic data sets, platform technologies, public investments, and multinational collaborations. Monetary contributions should therefore enter a pooled mechanism rather than depend on sequence-by-sequence causation [8,9]. Pooling should still recognise that countries contribute materials and sequence data, surveillance capacity, outbreak detection, laboratory infrastructure, and the recurrent costs of maintaining sharing systems.

For prospective testing, the allocation rule should be published before an emergency and give greater weight to need than to ownership claims. For scenario testing rather than as a normative recommendation, one illustrative starting formula would allocate 40% of the score to current public-health need, 25% to structural vulnerability, 20% to documented PABS contributions, and 15% to laboratory, regulatory, or manufacturing-capacity gaps. A technical panel could calculate scores from predefined indicators, with the Conference of the Parties approving the methodology and periodically reviewing the weights. This is a transparent starting hypothesis, not a settled formula.

Administrative challenges to scoring or eligibility could first be referred to an independent review panel, while disputes between parties concerning interpretation or application of the Pandemic Agreement would remain subject to Article 23 [1]. Contract disputes with manufacturers should follow the governing-law and dispute-resolution clauses in the standard agreement. This separates technical allocation questions, inter-state disputes, and private contractual claims.

## TESTING POLITICAL FEASIBILITY IN PABS NEGOTIATIONS

Technical coherence does not guarantee political acceptance. Pathogen-providing States may argue that a downstream model weakens sovereign control, delays benefits, or dilutes recognition through pooling. Manufacturers and research-intensive states may object that commercial triggers create uncertainty, compliance costs, confidentiality risks, or competitive disadvantages. Universities may resist rules that blur public research and commercialisation. These objections cannot be resolved by design elegance alone.

The proposal addresses these concerns through a limited bargain: routine scientific access remains low-friction; trigger events are specified in advance; provenance need not prove sequence-level causation; contribution retains a defined role in pooled allocation; obligations are tiered; and confidential technical information is protected while aggregate compliance is visible. These features may reduce, but cannot eliminate, conflict. The model remains vulnerable if major manufacturers stay outside PABS or provider states judge pooled benefits insufficiently connected to their contributions.

The framework should therefore be treated as a testable governance proposal rather than a near-complete solution. Member States could use legal mapping, contract simulation, and outbreak tabletop exercises to test trigger definitions, pass-through clauses, auditability, allocation weights, and non-participants. Post-entry review could revise thresholds or allocation rules in light of implementation evidence.

## CONCLUSIONS

The PABS annex remained under negotiation after the seventh IGWG meeting, with further negotiations scheduled before the Eightieth World Health Assembly [7,13]. The downstream model proposed here is one possible way to operationalise reciprocity, not an established solution. It could preserve rapid scientific access while making benefit-sharing obligations more predictable at defined points of product-oriented or commercial use. Its credibility depends on unresolved questions of legal authority, national-law alignment, participation incentives, pass-through enforcement, allocation governance, and political acceptance. These questions should be negotiated explicitly and tested prospectively. A durable bargain would combine speed with enforceable reciprocity while retaining the capacity to revise rules when evidence shows that the balance is not working.

## Data Availability

**Data availability:** No data sets were generated or analysed for this viewpoint. The manuscript is based on publicly available documents and published literature.

## References

[1] World Health Organization. WHO Pandemic Agreement. Resolution WHA78.1. Geneva, Switzerland: World Health Organization; 2025. Available: https://apps.who.int/gb/ebwha/pdf_files/WHA78/A78_R1-en.pdf. Accessed: 12 July 2026.

[2] World Health Organization. Achieving 70% COVID-19 immunization coverage by mid-2022. Geneva, Switzerland: World Health Organization; 2021. Available: https://www.who.int/news/item/23-12-2021-achieving-70-covid-19-immunization-coverage-by-mid-2022. Accessed: 12 July 2026.

[3] Wouters OJ Shadlen KC Salcher-Konrad M Pollard AJ Larson HJ Teerawattananon Y Challenges in ensuring global access to COVID-19 vaccines: production, affordability, allocation, and deployment. Lancet. 2021;397:1023–34. 10.1016/S0140-6736(21)00306-8 33587887 PMC7906643

[4] Gostin LO Halabi SF Klock KA An international agreement on pandemic prevention and preparedness. JAMA. 2021;326:1257–8. 10.1001/jama.2021.16104 34524388

[5] Secretariat of the Convention on Biological Diversity. Convention on Biological Diversity: text and annexes. Montreal, Canada: Secretariat of the Convention on Biological Diversity; 2011. Available: https://www.cbd.int/doc/legal/cbd-en.pdf. Accessed: 6 November 2026.

[6] Secretariat of the Convention on Biological Diversity. Nagoya Protocol on Access to Genetic Resources and the Fair and Equitable Sharing of Benefits Arising from their Utilization to the Convention on Biological Diversity: text and annex. Montreal, Canada: Secretariat of the Convention on Biological Diversity; 2011. Available: https://www.cbd.int/abs/doc/protocol/nagoya-protocol-en.pdf. Accessed: 6 November 2026.

[7] World Health Organization. Report of the seventh meeting of the open-ended Intergovernmental Working Group on the WHO Pandemic Agreement. Document A/IGWG/7/4. Geneva, Switzerland: World Health Organization; 2026. Available: https://apps.who.int/gb/IGWG/pdf_files/IGWG7/A_IGWG7_4-en.pdf. Accessed: 19 August 2026.

[8] Berman A Van Vooren B Access and benefit sharing: streamlining legal frameworks for global health equity. BMJ Public Health. 2025;3:e002830. 10.1136/bmjph-2025-002830 40166607 PMC11956343

[9] Sett S Kress WJ Halewood M Nicholson D Nuñez-Vega G Faggionato D Harmonize rules for digital sequence information benefit-sharing across UN frameworks. Nat Commun. 2024;15:8745. 10.1038/s41467-024-52994-z 39384744 PMC11464523

[10] Black A MacCannell DR Sibley TR Bedford T Ten recommendations for supporting open pathogen genomic analysis in public health. Nat Med. 2020;26:832–41. 10.1038/s41591-020-0935-z 32528156 PMC7363500

[11] World Health Organization. Pandemic influenza preparedness framework: sharing of influenza viruses and access to vaccines and other benefits. Geneva, Switzerland: World Health Organization; 2011. Available: https://www.who.int/publications/i/item/9789241503082. Accessed: 12 July 2026.

[12] Buck M Hamilton C The Nagoya Protocol on access to genetic resources and the fair and equitable sharing of benefits arising from their utilization to the Convention on Biological Diversity. Rev Eur Community Int Environ Law. 2011;20:47–61. 10.1111/j.1467-9388.2011.00703.x

[13] World Health Organization. Working modalities and updated timeline and deliverables of the open-ended Intergovernmental Working Group. Document A/IGWG/7/3 Rev.1. Geneva, Switzerland: World Health Organization; 2026. Available: https://apps.who.int/gb/IGWG/pdf_files/IGWG7/A_IGWG7_3Rev1-en.pdf. Accessed: 19 August 2026.

